# Specific regulations of gill membrane fatty acids in response to environmental variability reveal fitness differences between two suspension-feeding bivalves (*Nodipecten subnodosus* and *Spondylus crassisquama*)

**DOI:** 10.1093/conphys/coaa079

**Published:** 2020-08-25

**Authors:** Margaux Mathieu-Resuge, Fabienne Le Grand, Gauthier Schaal, Salvador E Lluch-Cota, Ilie S Racotta, Edouard Kraffe

**Affiliations:** 1 Univ Brest, CNRS, IRD, Ifremer, LEMAR, IUEM, F-29280, Plouzane, France; 2 WasserCluster Lunz—Inter-University Centre for Aquatic Ecosystem Research, Dr. Carl Kupelwieser Promenade 5, A-3293, Lunz am See, Austria; 3 Centro de Investigaciones Biológicas del Noroeste (CIBNOR), La Paz, BCS, Mexico

**Keywords:** Bivalves, coastal lagoon, gill membrane fatty acids, diet influence, physiological regulation, trophic ecophysiology

## Abstract

Bivalves’ physiological functions (i.e. growth, reproduction) are influenced by environmental variability that can be concomitant with trophic resource variations in terms of quality and quantity. Among the essential molecules that bivalves need to acquire from their diet to maintain physiological functions, fatty acids (FAs) such as polyunsaturated fatty acids (e.g. 20:4n-6 (arachidonic acid), 20:5n-3 (eicosapentaenoic acid) and 22:6n-3 (docosahexaenoic acid)) have been described to play a critical role. The present study examined the FA composition of gill membrane lipids of two bivalve species, *Nodipecten subnodosus* and *Spondylus crassisquama*, sampled in a coastal lagoon of the Northeastern Pacific (Ojo de Liebre, Mexico), at two contrasting locations (inner versus outer part of the lagoon) and at two different periods (February and August 2016). Spatiotemporal variations showed that FA composition of gill membrane lipids was highly correlated to FA composition of reserve lipids from digestive gland. This highlights the marked impact of the diet on FA composition of gill membranes. Interestingly, both species presented differences in the seasonal accumulations of plasmalogens and of particular FA that are not found in their diet (e.g. non-methylene interrupted FA, 22:4n-9*trans*, 20:1n-11), suggesting specific regulations of FA incorporation and lipid class composition in gill membranes to maintain optimal membrane function in their specific and changing environment. This study highlights the importance to characterize the spatial and temporal variability of food resources in order to apprehend the physiological consequences of environmental variability, as well as species differential regulation capacities in a changing world.

## Teaser

Gill membrane lipids of two bivalves presented specific regulations in response to both environmental and dietary resource variabilities. This highlights the importance of considering both trophic and physiological processes when studying responses to environmental stressors; an approach that can be defined as ‘trophic ecophysiology’.

## Introduction

The fatty acid (FA) composition of poikilotherms cell membranes is important for the maintenance of cell functions and properties, hence for proper organism health status (Kraffe *et al.*, 2004; Pernet *et al.*, 2007; Le Grand *et al.*, 2013). In particular, the cell membrane functioning and the organism capacity to cope with temperature changes are highly dependent on the proportion of polyunsaturated fatty acids (PUFAs) (Delaporte *et al.*, 2005; Pernet *et al.*, 2007; Parent *et al.*, 2008). Marine animals have limited capacities of *de novo* biosynthesis of PUFA and largely rely on dietary inputs (Arts *et al.*, 2001; Hulbert *et al.*, 2014). Once assimilated by animals, dietary FA can either be incorporated in cell membrane composition (polar lipids, PLs) as phospholipids or be stored in reserve tissues (neutral lipids, NLs), mostly as triglycerides (Arts *et al.*, 2001; Martin-Creuzburg *et al.*, 2012; Hulbert *et al.*, 2014). Three essential FAs (EFAs) are recognized to be crucial for cell membrane functions: arachidonic acid (ARA) 20:4n-6, eicosapentaenoic acid (EPA) 20:5n-3 and docosahexaenoic acid (DHA) 22:6n-3 (Delaporte *et al.*, 2005). For sessile or limited mobility species, dietary FA inputs can drastically change in quantity and quality at different temporal and spatial scales, and therefore the species’ capacities to store FA and/or to maintain an optimal cell membrane FA composition may influence their ability to cope with physiological requirements under a changing environment.

Frequently, marine suspension-feeding bivalves inhabiting coastal areas face strong variations in the environment (T°C, salinity, oxygen, etc.) and quantity and quality of food sources (phytoplankton, detritus, zooplankton, etc.) at various spatial and temporal scales, affecting all levels of organization within the organism. Responses at the cell level are essential to maintain physiological functions (i.e. growth, reproduction). In sub-Arctic and temperate environments, one of the strategies displayed by bivalves to cope with their physiological requirements when facing environmental variability is maintaining high long-chain PUFA concentrations in cell membrane during the coldest period of the year (Hazel and Williams, 1990; Delaporte *et al.*, 2005; Hayward *et al.*, 2007; Pernet *et al.*, 2007). Although the biosynthesis of PUFA in bivalves is limited, and most of these FA have to be acquired from their food sources, some particular unsaturated FA can be biosynthesized *de novo* by marine bivalves and can play key role in the maintenance of cell functionality under stressful conditions. These FAs, such as non-methylene interrupted (NMI) FA, 22:4n-9*trans* and 20:1n-11, are specifically associated to particular membrane phospholipids called plasmalogens (Kraffe *et al.*, 2004, 2006; Le Grand *et al.*, 2011, 2014). The functional role of these molecular species (plasmalogens and *de novo* biosynthesized FA) is still poorly understood. Because of their high concentration in the cell membrane, several studies have suggested that they may be playing an important role in the membrane structure function of different species of bivalve (Kraffe *et al.*, 2004; Delaporte *et al.*, 2005; Pernet *et al.*, 2007; Munro and Blier, 2012). Considering their implication in response to concomitant dietary changes in FA inputs and changes of others environmental factors appears to be essential.

The coastal lagoon of Ojo de Liebre (Baja California Sur, Mexico) is a 450 km^2^ enclosed water body facing the southern California Current area. The lagoon is protected as a UNESCO world heritage site and a Ramsar wetland site, in particular as a reproduction and wintering site for a diversity of marine mammals, birds and sea turtles. Besides its critical ecological role, the lagoon did host until 2010 one of the main bivalve fisheries of Mexico, targeting the pectinid *Nodipecten subnodosus* (G.B. Sowerby I, 1835). The fishery reached an historical maximum of 157 annual tons of scallop meat in 1999. The management strategy appeared successful and production remained nearly constant for a decade. However, in 2010, the stock started to collapse due to still undetermined causes, leading to the closure of the fishery in 2012. Although some hypotheses including parasite infections potentially linked to a poor health condition have been suggested (Ruiz-Verdugo *et al.*, 2016), there is still no satisfactory explanation to stocks collapse. Since 2012, the fishing effort has been diverted towards the spondylid *Spondylus crassisquama* (Lamarck, 1819), whose population within the lagoon seems to have strongly increased over the past decade, with an estimated population of >20 million individuals in the lagoon (Centro Regional de Investigación de Pesquera and Comisión Nacional de Acuacultura y Pesca, pers comm.).

Previous studies investigating the trophic ecology of *S. crassisquama* within the lagoon of Ojo de Liebre evidenced the spatial and temporal heterogeneity of dietary inputs within the lagoon (Mathieu-Resuge *et al.*, 2019a, 2019b). The vicinity of the lagoon’s entrance was characterized by high diatoms and associated EPA availability due to oceanic inputs in summer. The FA composition of NL and potential food sources (i.e. particulate organic matter and sediment organic matter) from the inner lagoon displayed lower amounts of this PUFA, and higher concentrations in C18 PUFA. The opposite pattern was observed in winter, where DHA and C18 PUFA were dominant near the mouth, while EPA dominated dietary inputs in the inner lagoon. Environmental variability in the lagoon is characterized by temperatures ranging from 18.6°C in winter to 24.4°C in summer at the entrance of the lagoon, and from 19.4°C to 25.6°C at the inner part of the lagoon (pers. comm., Reserva de la Biosfera El Vizacaino). Dissolved oxygen present annual variations of 0.8 mg 0_2_ L^−1^ at the entrance of the lagoon, and of 1.1 mg 0_2_ L^−1^ at the inner part of the lagoon between summer and winter. The contrasting FA inputs in the diet of bivalves, associated with temporally variable abiotic conditions, suggest that the ability of bivalves to regulate their cell membrane FA composition might contribute to the ecological success of the different species.

**Figure 1:**
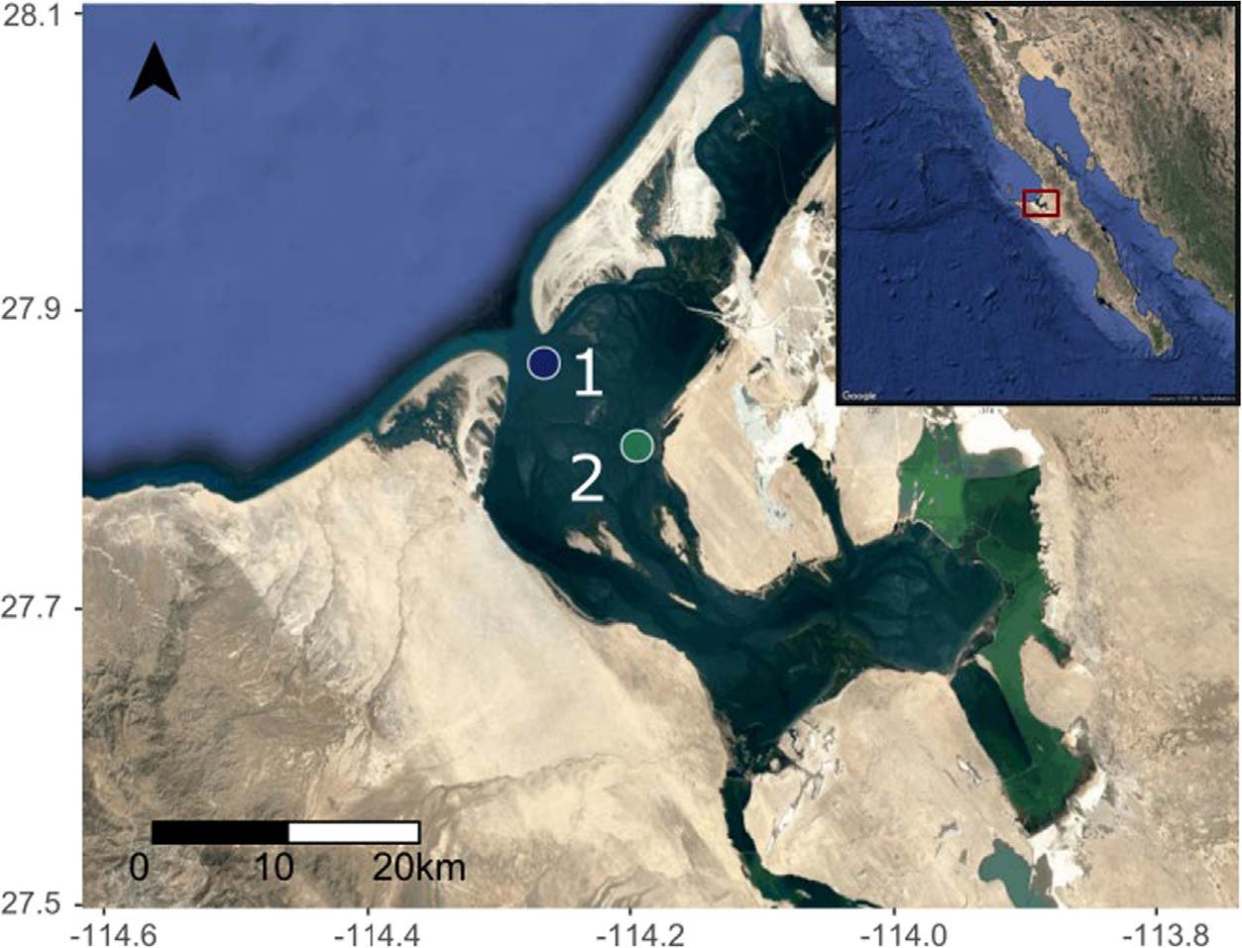
Location of the two sampling stations in the Ojo de Liebre lagoon (Baja California Sur, Mexico).

This work contributes to the understanding of the biological processes underlying the shift in species ecological dominance within the Ojo de Liebre bivalve assemblages, by evaluating the regulation capacities of the cell membrane FA composition in response to contrasting trophic and abiotic conditions for the two bivalve species *S. crassisquama* and *N. subnodosus*. It has been hypothesized that the two species would display different regulation capacities, which could have contributed to the recent evolution of their stocks. To test this assumption, individuals of both species were sampled from two locations within the lagoon, at two distinct periods (February and August) characterized by contrasting environmental and trophic conditions. Gills were used as the tissue representing physiological regulation of FA composition at cell membrane level and compared to the FA composition of reserve lipids of digestive gland (DG), representing the reserve tissue.

## Materials and methods

### Sampling

Sampling was realized in February and August 2016 at two stations, located on historically fished banks with contrasted distances to the mouth of the bay (Fig. 1) and characterized by depth ranging from 7 to 10 m. Station 1 is a bank located 6.1 km from the entrance of the lagoon and is considered as the ‘entrance site’, while station 2 is situated 15.5 km of the mouth and is considered as the ‘inner site’. Station 2 represents the southern distribution limit of bivalve species in the lagoon. Both stations were characterized by annual variations of abiotic conditions. Temperatures recorded at station 1 and 2 showed that mean temperatures were higher in August 2017 (24.4 ± 0.4°C and 25.6 ± 1.2°C, respectively) than in January 2018 (18.6 ± 0.4°C and 19.4 ± 1.3°C, respectively). The dissolved oxygen was ranging between 6.6 ± 0.5 mg 0_2_ L^−1^ in August and 7.4 ± 0.2 mg 0_2_ L^−1^ in January at station 1, and between 7.4 ± 1.6 mg 0_2_ L^−1^ and 8.5 ± 0.9 mg 0_2_ L^−1^ at station 2 (pers. comm., Reserva de la Biosfera El Vizacaino).

At each station and each sampling date, 10 individuals of *N. subnodosus* and *S. crassisquama* (length between 9 and 11 cm) were extracted by professional hookah divers. Bivalves were kept in cooler boxes filled with lagoon water for transportation to the local laboratory (CIBNOR, Unidad Guerrero Negro), where gills and DG were immediately dissected, packed and stored in liquid nitrogen for transportation to the main laboratory (CIBNOR, La Paz) where samples were then stored at −80°C until lipid extractions.

### Fatty acid analysis

#### Lipid extraction

Bivalve gills and DG were ground by ball milling under liquid nitrogen into a fine and homogeneous powder. The lipid extractions were carried out following a modified method by Folch *et al.* (1957). Briefly, 0.2 to 0.4 g of tissue powder was put in glass tubes containing 6 ml of chloroform/methanol (2,1; v/v) and then sonicated 5 min at 4°C. Lipid extracts were subsequently stored at −20°C under nitrogen atmosphere until further analysis.

#### Separation of neutral and polar lipids

Samples of total lipids were divided into two fractions according to the method described by Le Grand *et al.* (2014). One ml of gills and DG lipid extracts was evaporated to dryness, and lipids were recovered with three washings of 0.5 ml of chloroform/methanol (98:2, v/v) and deposited at the top of a silica gel micro-column (40 mm × 4 mm, silica gel 60A 63–200 μm rehydrated with 6% H_2_O (70–230 mesh)). NLs (corresponding to reserve lipids) were eluted with 10 ml of chloroform/methanol (98/2; v/v). The PL fraction (membrane lipids) was recovered with 20 ml of methanol. Both fractions were stored at−20°C for further FA analysis composition by gas chromatography. In each fraction, 2.3 μg of an internal standard (tricosanoic acid (C23:0)) was added.

#### FA analysis by gas chromatography

NL fraction from DG and PL fraction from gills were first evaporated to dryness under nitrogen and transesterified for 10 min at 100°C after adding 0.8 ml of methanol/H_2_SO_4_ (3.4%; v/v) following a modified methods by Le Grand *et al.* (2014). This method results in the formation of FA methyl esters (FAME) and dimethylacetals (DMAs) respectively obtained from the acyl chains (acyl-lipids, e.g. diacylphospholipids, triglycerides) and alkenyl chains (plasmalogen sn-1 chain) of lipids. DMA quantification allowing thus the indirect quantification of plasmalogens. After cooling and adding 0.8 ml of hexane, the organic phase containing FAME and DMA was washed three times with 1.5 ml of hexane-saturated water. At each washing, vials were shaken and centrifuged (1 min at 738 g; 20°C), and aqueous phase (containing glycerol, water and acid) was eliminated.

Both FAME and DMA from NL and PL fractions were analyzed on a Varian CP8400 gas chromatograph, equipped with two splitless injectors programmed at 220°C, and two flame-ionization detectors programmed at 280°C, with hydrogen as vector gas. The program in temperature was from 0°C to 150°C at 50°C min^−1^, then to 170°C at 3.5°C min^−1^, to 185°C at 1.5°C min^−1^, to 225°C at 2.4°C min^−1^ and finally to 250°C at 5.5°C min^−1^ and maintained for 15 min. To avoid coelution issues, FAME and DMA were separated simultaneously on two columns presenting different polarities, one polar (ZBWAX: 30 m × 0.25 mm ID × 0.2 μm, Phenomenex) and one apolar (ZB5HT: 30 m × 0.25 mm ID × 0.2 μm, Phenomenex). Both FAME and DMA were identified by comparing their retention time with references from commercial and in-house standard mixtures from marine bivalves. FA relative proportions were expressed as mass percentages of the total FA content. The unsaturation index (UI) was calculated according to the following equation (see below),

in which ∑MUFA is defined as the total% of unsaturated FA with one double bond and ∑nUFA as the total% of unsaturated FA with n double bonds. 

The FA compositions of the NL of the DG of *S. crassisquama* have already been characterized in a previous

$$\qquad\qquad\qquad UI=\frac{\sum \mathrm{MUFA}\ \mathrm{x}\ 1+\sum 2\mathrm{UFA}\ \mathrm{x}\ 2+\sum 3\mathrm{UFA}\ \mathrm{x}\ 3+\sum 4\mathrm{UFA}\ \mathrm{x}\ 4\sum 5\mathrm{UFA}\ \mathrm{x}\ 5+\sum 6\mathrm{UFAx}6\ }{100.}\quad \quad \quad $$

study concerning the trophic ecology of this bivalve species (Mathieu-Resuge *et al.*, 2019a). For better readability, results are presented in Table 3 next to those of *N. subnodosus*.

### Statistical analysis

Due to the number of zeros in data sets that was always inferior to 2.5%, Euclidean distance was applied in all statistical analyses. Only FAs accounting for >0.5% at one station and/or period were considered for the statistical analysis. Normality (Shapiro–Wilks test) and homoscedasticity (Bartlett test) were tested. For all data, conditions were not satisfied, and non-parametric analyses were therefore carried out.

The difference of the global PL composition of gills between periods (February and August) was tested by permutational variance analysis (PERMANOVA). In order to compare the seasonal fluctuation of the abundance of gill PL and DG NL, each FA of interest (i.e. accounting for >0.5%) was tested by a PERMANOVA (Anderson, 2014), and this is for both species at each station. The correlations between particular EFA content in each tissues were tested using Spearman’s rank correlation (where *r*_s_ is the correlation coefficient). When a correlation was detected, a linear model was fitted adjusted and the normality of residuals was checked using Q-Q plots. All statistical analyses and graphics were performed with the free software R (Core Team, 2017), with R Version 3.4.1 (2017 06 30).

**Table 1:** Polar lipid FA composition (mean ± SD; mass %; *n* = 10) of *Nodipecten subnodosus* gills in February and August, for both stations. Only the FAs accounting for >1% of total FA in at least one sample are shown. Different letters indicate significant difference (significant level α < 0.05) of seasonal variations tested by PERMANOVA at stations 1 and 2, respectively.

	**Station 1**						**Station 2**					
	**February**			**August**			**February**			**August**		
**∑ BRANCHED** ^a^	**0.6**	**±0.1**	**b**	**0.9**	**±0.0**	**a**	**0.6**	**±0.1**	**B**	**1.2**	**±0.1**	**A**
14:0	0.7	±0.1	**b**	1.4	±0.2	**a**	0.9	±0.1	**B**	1.4	±0.2	**A**
16:0	8.5	±0.9	**b**	11.3	±0.6	**a**	9.1	±0.3	**B**	14.0	±1.4	**A**
17:0	1.4	±0.2		1.5	±0.1		1.6	±0.1		1.7	±0.2	
18:0	8.7	±0.8	**a**	6.5	±0.5	**b**	9.2	±0.4	**A**	7.0	±0.4	**B**
20:0	3.1	±0.6		3.4	±0.2		2.3	±0.1	**B**	3.1	±0.4	**A**
**∑ SFA**	**23.9**	**±1.3**	**b**	**25.7**	**±0.9**	**a**	**24.6**	**±0.8**	**B**	**29.0**	**±2.5**	**A**
16:1n-9	0.3	±0.1		0.2	±0.0		0.5	±0.0	**A**	0.3	±0.0	**B**
16:1n-7	0.6	±0.2	**b**	1.8	±0.1	**a**	0.5	±0.0	**B**	1.2	±0.1	**A**
16:1n-5	0.8	±0.4	b	2.9	±0.3	a	0.2	±0.1	**B**	2.9	±0.5	A
18:1n-9	1.8	±0.2		1.6	±0.8		1.5	±0.2	**B**	4.3	±0.5	**A**
18:1n-7	0.9	±0.1	**b**	2.0	±0.1	**a**	1.1	±0.1	**B**	2.0	±0.3	**A**
20:1n-11	1.3	±0.1	**b**	2.0	±0.1	**a**	1.2	±0.1	**B**	1.6	±0.2	**A**
20:1n-9	2.0	±0.2	**a**	1.7	±0.1	**b**	1.9	±0.2		1.9	±0.3	
**∑ MUFA**	**9.6**	**±0.7**	**b**	**14.5**	**±0.8**	**a**	**9.3**	**±0.3**	**B**	**15.6**	**±0.7**	**A**
16:3n-4	0.8	±0.1	**a**	0.5	±0.1	**b**	0.4	±0.0		0.4	±0.0	
18:2n-6	0.5	±0.1	**b**	0.6	±0.0	**a**	0.5	±0.0	**B**	1.3	±0.1	**A**
18:3n-3	0.5	±0.1	**a**	0.4	±0.0	**b**	0.5	±0.0	**B**	1.2	±0.1	**A**
18:4n-3	0.5	±0.1	**a**	0.3	±0.0	**b**	0.6	±0.1	**B**	0.9	±0.1	**A**
20:4n-6 (ARA)	6.2	±0.9	**b**	7.4	±0.2	**a**	5.4	±0.3		5.2	±0.7	
20:5n-3 (EPA)	4.8	±0.4	**b**	5.1	±0.3	**a**	5.9	±0.3		5.6	±0.5	
22:4n-6	2.4	±0.2	**b**	2.9	±0.2	**a**	1.9	±0.2	**A**	1.3	±0.2	**B**
22:4n-9*trans*	5.9	±0.6	**a**	4.2	±0.3	**b**	6.1	±0.4	**A**	3.2	±0.5	**B**
22:5n-6	2.7	±0.2	**a**	2.1	±0.1	**b**	2.5	±0.1	**A**	2.1	±0.1	**B**
22:5n-3	0.9	±0.1	**a**	0.8	±0.0	**b**	1.0	±0.1	**A**	0.8	±0.1	**B**
22:6n-3 (DHA)	20.5	±1.1	**a**	14.2	±0.7	**b**	20.0	±0.6	**A**	14.6	±0.9	**B**
**∑ PUFA**	**52.1**	**±1.4**	**a**	**44.8**	**±1.0**	**b**	**51.5**	**±0.8**	**A**	**42.5**	**±2.1**	**B**
16:0DMA	0.9	±0.1		0.9	±0.1		1.1	±0.1		1.0	±0.2	
16:1n-7DMA	1.3	±0.7	**b**	3.9	±0.3	**a**	0.5	±0.2	**B**	3.7	±0.6	**A**
17:0DMA	1.0	±0.1	**a**	0.8	±0.1	**b**	1.1	±0.1	**A**	0.7	±0.1	**B**
18:0DMA	7.9	±0.7	**a**	6.2	±0.5	**b**	7.5	±0.7	**A**	4.2	±0.6	**B**
**∑ DMA**	**11.7**	**±0.7**		**12.0**	**±0.3**		**11.6**	**±0.6**	**A**	**9.7**	**±0.9**	**B**
20:2i (5:11)	0.9	±0.0	**a**	0.8	±0.1	**b**	1.0	±0.1		1.1	±0.1	
22:2i (7:13)	1.6	±0.1	**a**	0.9	±0.1	**b**	1.4	±0.2	**A**	1.0	±0.1	**B**
**∑ NMI** ^b^	**4.1**	**±0.2**	**a**	**3.3**	**±0.1**	**b**	**4.3**	**±0.3**	**A**	**3.2**	**±0.3**	**B**
**∑ UNKNOWN**	**1.0**	**±0.1**	**b**	**1.1**	**±0.1**	**a**	**1.2**	**±0.1**		**1.2**	**±0.1**	
**∑** n-3	27.9	±1.4	**a**	21.4	±0.6	**b**	28.9	±0.9	**A**	23.6	±1.3	**B**
**∑** n-6	13.9	±1.3	**b**	15.7	±0.4	**a**	12.3	±0.4		12.3	±0.9	
UI^c^	2.6	±0.07	**a**	2.3	±0.0	**b**	2.6	±0.0	**A**	2.2	±0.1	**B**

SFA, saturated FA; MUFA, monosaturated FA; PUFA, polyunsaturated FA; DMA, dimethylacetals; Unknown, unidentified FA.

^a^Branched: FA (sum of iso15:0, anteiso15:0, iso16:0, iso17:0 and anteiso:017).

^b^NMI: non-methylene interrupted FA (sum of 20:2i (5:11), 20:2j (5:13), 20:3 nmi, 22:2i (7:13), 22:2j (7:15), 22:3 nmi)**.**

^c^Unsaturation index: calculated according to the equation [∑MUFA × 1 + ∑2UFA × 2 + ∑3UFA × 3 + ∑4UFA × 4 ∑5UFA × 5 + ∑6UFA × 6]/100.

## Results

Both spatial and temporal differences were found in the FA composition of gill membrane lipids of the two species. Because significant interactions were found between sampling dates (February and August) and stations (1 and 2) for *N. subnodosus* (PERMANOVA, *P* value < 0.001, *df* = 1, *F* = 11.32, *R*^2^ = 0.05) and *S. crassisquama* (PERMANOVA, *P* value < 0.05, *df* = 1, *F* = 4.51, *R*^2^ = 0.02), we compared the difference between sampling dates at each station (Tables 1 and 2). At both stations, we observed significant different global FA composition of gills between sampling time, for both *N. subnodosus* (PERMANOVA, *P* value < 0.001, *df* = 1, *F* = 75.8, *R*^2^ = 0.81 for station 1, *P* value < 0.001, *df* = 1, *F* = 97.5, *R*^2^ = 0.84 for station 2) and *S. crassisquama* (PERMANOVA, *P* value < 0.001, *df* = 1, *F* = 81.8, *R*^2^ = 0.83 for station 1, *P* value < 0.001, *df* = 1, *F* = 74.5, *R*^2^ = 0.81 for station 2). Same significant difference of DG global FA composition was observed between sampling time at each station, for both *N. subnodosus* (PERMANOVA, *P* value < 0.001, *df* = 1, *F* = 69, *R*^2^ = 0.8 for station 1, *P* value < 0.001, *df* = 1, *F* = 21.6, *R*^2^ = 0.55 for station 2) and *S. crassisquama* (PERMANOVA, *P* value < 0.001, *df* = 1, *F* = 6.55, *R*^2^ = 0.27 for station 1, *P* value < 0.001, *df* = 1, *F* = 45.9, *R*^2^ = 0.72 for station 2).

**Table 2:** Polar lipid FA composition (mean ± SD; mass %; *n* = 10) of *Spondylus crassisquama* gills in February and August, for both stations. Only the FAs accounting for >1% of total FA in at least one sample are shown. Different letters indicate significant difference (significant level α < 0.05) of seasonal variations tested by PERMANOVA at stations 1 and 2, respectively.

	**Station 1**						**Station 2**					
	**February**			**August**			**February**			**August**		
**∑ BRANCHED** ^a^	**1.1**	**±0.1**	**b**	**1.5**	**±0.2**	**a**	**0.9**	**±0.1**	**B**	**1.8**	**±0.2**	**A**
14:0	0.7	±0.0	**b**	1.4	±0.2	**a**	0.8	±0.1	**B**	1.2	±0.2	**A**
16:0	6.3	±0.5	**b**	8.2	±0.6	**a**	7.0	±0.7	**B**	8.6	±1.0	**A**
17:0	1.5	±0.3		1.6	±0.1		2.0	±0.2	**A**	1.7	±0.1	**B**
18:0	6.6	±1.4		7.2	±0.5		7.7	±0.6		7.2	±0.5	
20:0	2.5	±0.2		2.5	±0.4		2.5	±0.2		2.3	±0.3	
**∑ SFA**	**18.9**	**±2.4**	**b**	**22.5**	**±1.0**	**a**	**21.7**	**±1.6**		**22.9**	**±1.4**	
16:1n-9	0.9	±0.0	a	0.2	±0.0	**b**	0.9	±0.1	**A**	0.3	±0.1	**B**
16:1n-7	0.4	±0.0	**b**	2.3	±0.3	**a**	0.5	±0.1	**B**	1.5	±0.1	**A**
16:1n-5	0.3	±0.0	**b**	2.4	±0.4	a	0.3	±0.0	**B**	2.3	±0.5	**A**
18:1n-9	1.3	±0.1	**b**	1.7	±0.3	**a**	1.1	±0.1	**B**	2.6	±0.3	**A**
18:1n-7	1.0	±0.0	**b**	2.0	±0.2	**a**	1.1	±0.0	**B**	1.5	±0.1	**A**
20:1n-11	3.2	±0.1	**b**	5.7	±0.2	**a**	3.0	±0.2	**B**	6.3	±0.6	**A**
20:1n-9	1.9	±0.2	**b**	2.2	±0.2	**a**	1.8	±0.1	**B**	2.1	±0.4	**A**
**∑ MUFA**	**11.2**	**±0.3**	**b**	**18.2**	**±0.7**	**a**	**11.3**	**±0.3**	**B**	**18.1**	**±0.9**	**A**
16:3n-4	1.0	±0.1	**a**	0.4	±0.1	**b**	0.7	±0.1	**A**	0.5	±0.1	**B**
18:2n-6	0.8	±0.0	**b**	1.4	±0.2	**a**	0.9	±0.1	**B**	1.8	±0.2	**A**
18:3n-3	0.5	±0.0	**b**	0.6	±0.1	**a**	0.8	±0.1	**B**	1.3	±0.2	**A**
18:4n-3	0.6	±0.0	**a**	0.4	±0.1	**b**	0.8	±0.1		0.9	±0.2	
20:4n-6 (ARA)	8.2	±0.6	**b**	9.8	±0.7	**a**	6.6	±0.6		6.8	±0.6	
20:5n-3 (EPA)	4.5	±0.3		4.7	±0.6		6.3	±0.7	**A**	5.5	±0.8	**B**
22:4n-6	3.0	±0.2	**b**	3.4	±0.3	**a**	2.0	±0.2		1.7	±0.7	
22:5n-6	3.4	±0.2	**a**	2.5	±0.2	**b**	2.7	±0.2	**A**	2.4	±0.3	**B**
22:5n-3	0.7	±0.0	**a**	0.6	±0.2	**b**	0.8	±0.1	**A**	0.7	±0.1	**B**
22:6n-3 (DHA)	21.3	±1.1	**a**	14.8	±1.0	**b**	19.4	±0.9	**A**	13.8	±1.2	**B**
**∑ PUFA**	**48.7**	**±1.8**	**a**	**43.5**	**±1.9**	**b**	**45.4**	**±1.5**	**A**	**40.8**	**±1.1**	**B**
16:0DMA	1.1	±0.2	**a**	0.4	±0.1	**b**	1.2	±0.1	**A**	0.8	±0.2	**B**
16:1n-7DMA	0.5	±0.1	**b**	3.1	±0.5	**a**	0.5	±0.1	**B**	3.1	±0.6	**A**
17:0DMA	2.1	±0.1	**a**	0.7	±0.1	**b**	2.6	±0.1	**A**	1.1	±0.3	**B**
18:0DMA	8.9	±0.8	**a**	2.2	±0.5	**b**	8.5	±0.6	**A**	3.1	±1.3	**B**
**∑ DMA**	**13.1**	**±0.9**	**a**	**6.4**	**±1.0**	**b**	**13.2**	**±0.7**	**A**	**8.1**	**±1.4**	**B**
20:2i (5:11)	1.9	±0.1	**b**	2.3	±0.3	**a**	2.1	±0.2	**B**	2.8	±0.3	**A**
22:2i (7:13)	1.6	±0.1	**b**	1.9	±0.2	**a**	1.4	±0.2	**B**	2.1	±0.3	**A**
**∑ NMI** ^b^	**5.4**	**±0.2**	**b**	**6.4**	**±0.3**	**a**	**5.5**	**±0.3**	**B**	**6.9**	**±0.8**	**A**
**∑ UNKNOWN**	**1.7**	**±0.0**	**a**	**1.4**	**±0.1**	**b**	**2.1**	**±0.1**	**A**	**1.5**	**±0.1**	**B**
**∑** n-3	28.0	±1.2	**a**	21.4	±1.4	**b**	28.4	±1.0	**A**	22.7	±1.3	**B**
**∑** n-6	18.5	±1.0	**b**	20.5	±1.1	**a**	15.0	±0.8	**B**	16.3	±1.2	**A**
UI^c^	2.6	±0.1	**a**	2.3	±0.1	**b**	2.4	±0.1	**A**	2.2	±0.1	**B**

SFA, saturated FA; MUFA, monosaturated FA; PUFA, polyunsaturated FA; DMA, dimethylacetals; Unknown, unidentified FA.

^a^Branched: FA (sum of iso15:0, anteiso15:0, iso16:0, iso17:0 and anteiso:017).

^b^NMI: non-methylene interrupted FA (sum of 20:2i (5:11), 20:2j (5:13), 20:3 nmi, 22:2i (7:13), 22:2j (7:15), 22:3 nmi)**.**

^c^Unsaturation index: calculated according to the equation [∑MUFA × 1 + ∑2UFA × 2 + ∑3UFA × 3 + ∑4UFA × 4 ∑5UFA × 5 + ∑6UFA × 6]/100.

Total MUFA proportions of gills significantly increased from February to August, from 9.6% to 14.5% at station 1, and from 9.3% to 15.6% at station 2 for *N. subnodosus* (Table 1) and from 11.2% to 18.2% at station 1, and from 11.3% to 18.1% at station 2 for *S. crassisquama* (Table 2). Conversely, PUFA proportions of gills decreased significantly at both sites in August, with values from 52.1% to 44.8% at station 1 and from 51.5% to 42.5% at station 2 for *N. subnodosus* (Table 1), and from 48.7% to 43.5% at station 1 and from 45.4% to 40.8% at station 2 for *S. crassisquama* (Table 2). As a consequence, a significant decrease of the degree of unsaturation was found for both species in August at both stations (Tables 1 and 2).

### Seasonal changes of essential dietary FAs

**Figure 2:**
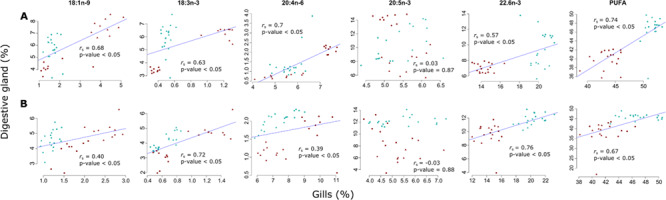
Spearman’s correlation for essential dietary FAs between digestive glands and gills of (A) *Nodipecten subnodosus* and (B) *Spondylus crassisquama*. The linear regression is represented when the *P* value is significant (α < 0.05). Individuals from station 1 are represented by square, those from station 2 by triangles. Samples from February are represented in blue, and those from August in red.

For both species, in August at station 2, gills were associated to significant higher levels of 18:1n-9 and 18:3n-3 (Tables 1 and 2). These differences between February and August were also found in NLs of DG of the same individuals (Table 3). However, these FA presented contrasted proportions between tissues: 18:1n-9 was twice as abundant in DG as in gills for both species, and 18:3n-3 was respectively four and five times more abundant in *S. crassisquama* and *N. subnodosus*. Pooled data showed that 18:1n-9 and 18:3n-3 proportions in gill membrane lipids were highly correlated to the proportions found in DG reserve lipids (*P* values < 0.05; Fig. 2). *N. subnodosus* showed a stronger positive correlation for 18:1n-9 (*r*_s_ = 0.68; Fig. 2) than *S. crassisquama* (*r*_s_ = 0.4; Fig. 2), while the opposite was found for 18:3n-3 (*r*_s_ = 0.63 and 0.72, for *N. subnodosus* and *S. crassisquama*, respectively; Fig. 2).

**Table 3:** Neutral lipids FA composition (mean ± SD; mass %; *n* = 10) of *Nodipecten subnodosus and Spondylus crassisquama* digestive glands in February and August, for both stations. Only the FAs accounting for >1% of total FA in at least one sample are shown. Different letters indicate significant difference (significant level α < 0.05) of seasonal variations tested by PERMANOVA at stations 1 and 2, respectively.

	*Nodipecten subnodosus*								*Spondylus crassisquama*							
	Station 1				Station 2				Station 1				Station 2			
	February		August		February		August		February		August		February		August	
**∑Branched** ^a^	**2.9**	**±1.3** ^**a**^	**1.4**	±**0.2**^**b**^	**1.7**	**±0.4**	**1.9**	±**0.3**	**1.4**	**±0.1**	**1.4**	**±0.2**	**1.3**	**±0.1** ^**b**^	**1.8**	**±0.4** ^**a**^
14:0	5.6	±0.6^**b**^	8.1	±0.7^**a**^	5.8	±0.7	5.8	±0.5	4.8	±0.4^**b**^	8.2	±1.5^**a**^	4.4	±0.3^**b**^	5.8	±0.9^**a**^
16:0	19.9	±0.8^**b**^	21.9	±0.5^**a**^	22.1	±2.4^**b**^	25.8	±1.1^**a**^	21.2	±0.6	21.1	±1.0	21.8	±1.3^**b**^	24.7	±1.3^**a**^
17:0	1.1	±0.3	1.0	±0.1	0.9	±0.1^**b**^	1.0	±0.1^**a**^	1.3	±0.1^**a**^	1.1	±0.2^**b**^	1.4	±0.1	1.3	±0.1
18:0	4.7	±1.7^**a**^	3.4	±0.3^**b**^	3.5	±0.7^**b**^	4.6	±0.4^**a**^	4.5	±0.3	4.3	±0.6	5.0	±0.7	5.2	±0.5
20:0	0.2	±0.1	0.2	±0.0	0.1	±0.0^**b**^	0.2	±0.0^**a**^	0.2	±0.0^**a**^	0.3	±0.1^**b**^	0.2	±0.0^**a**^	0.2	±0.0^**b**^
**∑SFA**	**32.6**	**±1.7** ^**b**^	**35.8**	±**0.8**^**a**^	**33.1**	**±3.4** ^**b**^	**38.5**	±**1.6**^**a**^	**33.0**	**±0.8** ^**a**^	**36.3**	**±2.0** ^**b**^	**33.7**	**±1.7** ^**b**^	**38.6**	**±2.0** ^**a**^
16:1n-9	0.5	±0.2^**a**^	0.3	±0.0^**b**^	0.5	±0.0	0.5	±0.0	0.4	±0.0	0.4	±0.1	0.3	±0.1^**b**^	0.6	±0.1^**a**^
16:1n-7	4.6	±0.5^**b**^	10.6	±0.5^**a**^	6.0	±0.6	5.6	±0.5	5.6	±0.3^**b**^	8.8	±0.9^**a**^	5.5	±0.4	5.2	±0.3
16:1n-5	0.1	±0.1^**b**^	0.3	±0.0^**a**^	0.2	±0.0^**b**^	0.2	±0.0^**a**^	0.3	±0.0	0.3	±0.0	0.2	±0.0	0.2	±0.0
18:1n-9	5.6	±1.3^**a**^	4.2	±0.4^**b**^	5.9	±0.7^**b**^	7.6	±0.7^**a**^	4.8	±0.5^**a**^	4.0	±0.5^**b**^	4.3	±0.6^**b**^	5.2	±0.6^**a**^
18:1n-7	2.3	±0.3^**b**^	3.1	±0.2^**a**^	2.7	±0.3	2.8	±0.2	2.2	±0.1	2.7	±0.3	2.7	±0.2	2.6	±0.1
20:1n-11	0.4	±0.1^**a**^	0.2	±0.0^**b**^	0.2	±0.1^**b**^	0.2	±0.1^**a**^	0.6	±0.1	0.4	±0.4	0.6	±0.1	0.7	±0.3
20:1n-9	0.7	±0.2^**a**^	0.5	±0.0^**b**^	0.6	±0.1^**b**^	0.7	±0.1^**a**^	0.7	±0.1	0.8	±0.3	0.8	±0.1^**b**^	1.2	±0.2^**a**^
**∑MUFA**	**15.3**	**±2.1** ^**b**^	**20.5**	±**0.4**^**a**^	**17.0**	**±0.6** ^**b**^	**19.0**	±**0.7**^**a**^	**16.3**	**±0.5** ^**b**^	**19.0**	**±0.5** ^**a**^	**16.3**	**±0.7** ^**b**^	**17.8**	**±0.5** ^**a**^
18:2n-6	3.2	±0.8	2.6	±0.1	3.3	±0.3^**b**^	3.7	±0.2^**a**^	2.7	±0.1^**a**^	2.5	±0.3^**b**^	2.9	±0.2	2.9	±0.1
18:3n-3	5.3	±1.2^**a**^	3.4	±0.2^**b**^	6.4	±0.7	6.3	±0.4	3.6	±0.1^**a**^	3.1	±0.4^**b**^	4.5	±0.4	4.8	±0.5
18:4n-3	9.7	±1.1^**a**^	5.0	±0.4^**b**^	9.8	±1.3^**a**^	7.4	±1.0^**b**^	5.7	±0.8^**a**^	4.8	±0.6^**b**^	5.7	±0.4	5.4	±0.8
20:4n-6 (ARA)	1.7	±1.0	2.1	±0.2	1.1	±0.1^**a**^	0.7	±0.1^**b**^	2.2	±0.1^**a**^	2.0	±0.2^**b**^	1.9	±0.2^**a**^	1.1	±0.2^**b**^
20:5n-3 (EPA)	8.9	±1.4^**b**^	14.2	±0.6^**a**^	11.7	±2.3^**a**^	7.8	±1.6^**b**^	11.9	±0.4^**a**^	10.8	±1.8^**b**^	12.5	±0.8^**a**^	6.7	±1.3^**b**^
22:4n-6	0.3	±0.2^**a**^	0.1	±0.0^**b**^	0.1	±0.0	0.1	±0.0	0.3	±0.0	0.4	±0.2	0.2	±0.0^**b**^	0.3	±0.1^**a**^
22:4n-9*trans*	0.4	±0.1^**a**^	0.2	±0.0^**b**^	0.3	±0.1	0.2	±0.0	NA		NA		NA		NA	
22:5n-6	0.6	±0.2^**a**^	0.4	±0.1^**b**^	0.4	±0.1	0.4	±0.0	0.8	±0.0^**b**^	0.7	±0.1^**a**^	0.7	±0.2^**b**^	0.9	±0.1^**a**^
22:5n-3	0.7	±0.2^**a**^	0.5	±0.0^**b**^	0.5	±0.0^**a**^	0.5	±0.1^**b**^	0.7	±0.0	0.8	±0.3	0.7	±0.0^**b**^	1.0	±0.2^**a**^
22:6n-3 (DHA)	11.6	±1.1^**a**^	7.4	±0.4^**b**^	7.7	±1.1^**a**^	6.7	±0.5^**b**^	12.5	±0.6^**a**^	10.2	±0.9^**b**^	11.3	±0.6^**a**^	9.6	±0.9^**b**^
**∑PUFA**	**47.9**	**±1.2** ^**a**^	**40.9**	±**1.2**	**46.5**	**±3.8** ^**a**^	**39.0**	±**2.3**^**b**^	**46.0**	**±0.9** ^**a**^	**40.6**	**±2.3** ^**b**^	**45.7**	**±1.8** ^**a**^	**38.4**	**±2.3** ^**b**^
**∑DMA**	**0.6**	**±0.1** ^**b**^	**0.8**	±**0.1**^**a**^	**0.9**	**±0.2**	**0.8**	±**0.1**	**2.5**	**±0.4** ^**a**^	**1.5**	**±0.3** ^**b**^	**2.2**	**±0.4**	**2.2**	**±0.5**
20:2i (5:11)	0.2	±0.1^**a**^	0.1	±0.0^**b**^	0.2	±0.1	0.1	±0.0	0.4	±0.1^**a**^	0.3	±0.1^**b**^	0.5	±0.1	0.5	±0.1
22:2i (7:13)	0.2	±0.1^**a**^	0.0	±0.0^**b**^	0.1	±0.0	0.1	±0.0	0.2	±0.0^**a**^	0.2	±0.1^**b**^	0.2	±0.0	0.2	±0.0
**∑NMI** ^b^	**0.6**	**±0.3** ^**a**^	**0.3**	**±0.0** ^**b**^	**0.3**	**±0.1**	**0.3**	**±0.1**	**0.8**	**±0.1** ^**a**^	**0.6**	**±0.2** ^**b**^	**0.9**	**±0.2** ^**a**^	**0.6**	**±0.1** ^**b**^
**∑Unknown**	**0.7**	**±0.1** ^**b**^	**0.6**	±**0.0**^**a**^	**0.8**	**±0.3**	**0.6**	±**0.0**	**0.8**	**±0.1** ^**b**^	**1.1**	**±0.3** ^**a**^	**0.8**	**±0.3** ^**b**^	**1.1**	**±0.2** ^**a**^

SFA, saturated FA; MUFA, monosaturated FA; PUFA, polyunsaturated FA; DMA, dimethylacetals; Unknown, unidentified FA.

^a^Branched: FA (sum of iso15:0, anteiso15:0, iso16:0, iso17:0 and anteiso:017).

^b^NMI: non-methylene interrupted FA (sum of 20:2i (5:11), 20:2j (5:13), 20:3 nmi, 22:2i (7:13), 22:2j (7:15), 22:3 nmi)**.**

Data from *Spondylus crassisquma* DG were previously published (Mathieu-Resuge *et al.*, 2019a) but were plotted in this table to facilitate interpretation of the present results for gill membrane FA composition.

For both species, levels of ARA in bivalve gills from station 1 were significantly higher in August than in February but did not vary at station 2 (Table 1 for *N. subnodosus*, and Table 2 for *S. crassisquama*). ARA proportions presented significant positive Spearman correlations between tissues (*P* values < 0.05, *r*_s_ = 0.7 and 0.39, for *N. subnodosus* and *S. crassisquama*, respectively; Fig. 2). Gills displayed significant lower levels of DHA in August than in February for both species and at both stations (Table 1 for *N. subnodosus* and Table 2 for *S. crassisquama*). Spearman correlations between gills and DG showed that lower levels of DHA found **in gills** of individuals sampled in August were correlated to lower levels of DHA in DG for both **species** considered (Fig. 2). Finally, if EPA presented important temporal variations in DG of both species (except for *S. crassisquama* at station 1; Table 3), these variations in gill membrane lipids were buffered. For *N. subnodosus*, only gills of individuals from station 1 presented higher levels of EPA in August (of only 0.3%; Table 1), and for *S. crassisquama*, only gill of individuals from station 2 showed significant higher levels of EPA in February (of 0.8%; Table 2). Indeed, in contrast to other EFA, no significant correlations were obtained for the proportion of EPA between tissues (*P* values = 0.87 and 0.88, for *N. subnodosus* and *S. crassisquama*, respectively; Fig. 2).

### Seasonal changes in plasmalogens-associated FAs

While temporal variations of total MUFA, PUFA and EFA were quite similar for both species, and at each station, some specific FA showed different variations depending of the period, the station and the species considered. This is particularly marked for plasmalogen-associated FA: NMI FA, 20:1n-11 and 22:4n-9trans. For *N. subnodosus*, total NMI FA proportion was significantly lower in August than in February at both sites (3.3% compared to 4.1% at station 1, and 3.2% compared to 4.3% at station 2; Table 1). The PUFA 22:4n-9*trans* in gills of *N. subnodosus* was also found in lower proportions in August (4.2% and 3.2% at stations 1 and 2), than in February (5.9% and 6.1% at stations 1 and 2; Table 1). The 20:1n-11 showed opposite seasonal fluctuations, with significant higher proportions in August than in February (2% compared to 1.3% at station 1, and 1.6% compared to 1.2% at stations 2; Table 1).

Contrary to *N. subnodosus*, the proportion of total NMI FA in *S. crassisquama* showed significant higher levels in August at both sites (6.4% compared to 5.4% at station 1, and 6.9% compared to 5.5% at station 2; Table 2). The MUFA 20:1n-11 followed trend of NMI FA and also increased significantly in August at both sites (from 3.2% to 5.7% at station 1, and from 3.0% to 6.3% at station 2; Table 2). The 22:4n-9 t was not detected in *S. crassisquama* gills membranes.

The proportion of total DMA in *N. subnodosus* gills did not vary significantly between periods at station 1 (11.7% in February and 12% in August, Table 1) but was significantly lower in August at station 2 showing values of 9.7%, compared to 11.6% in February (Table 1). Contrastingly, the amount of DMA in *S. crassisquama* gills significantly decreased in August at both stations (from 13.1% in February to 6.4% in August at station 1, and from 13.2% in February to 8.1% in August at station 2; Table 2).

## Discussion

This study shows strong and significant spatiotemporal variations in the FA composition of gill membrane lipids, which paralleled some of those observed for reserve lipids (i.e. dietary lipids) in DG, and highlights the role of dietary inputs in the FA composition of cell membranes. Nevertheless, differences in the relative concentrations of specific FA revealed the capacity of both species to selectively accumulate specific FA to meet their metabolic requirements. Such results provide evidence of cell membranes FA composition regulations. Taken together, although both species proved to be strongly affected by dietary inputs, the present study evidences the tradeoff between particular FA inputs and functional requirement in the lipid metabolism of marine bivalves.

### Comparison between dietary inputs and cell membranes FA composition

Once consumed, FAs are generally stored without important modifications as reserve lipids (mainly triacylglycerols) in reserve tissues (such as DG for marine bivalves) and mostly mirror their proportion in the diet. Their transfer into biological membranes (mainly as phospholipids) of functional tissues (i.e. gills) remains under metabolic control according to species and cells’ requirements (Arts *et al.*, 2001; Martin-Creuzburg *et al.*, 2012; Sardenne *et al.*, 2017).

A striking example was found at the inner lagoon station (station 2), for 18:1n-9 and 18:3n-3, where higher proportions in membrane lipids in individuals in August were correlated to higher proportion of these FA in reserve lipids of DG. Nevertheless, these FAs illustrate a first example of strong regulation and selective retention at the level of gill membranes since membrane lipids presented much lower levels than those found in reserve lipids. In contrast, for both species, EFA such as DHA and ARA were found in higher proportion in gills compared to DG. Bivalves are not able to biosynthesize *de novo* these FAs and are fully dependent on their diet (Arts *et al.*, 2001; Wacker *et al.*, 2002). The high proportions of these EFA in gills suggest the specific transfer from DG of these FA assimilated from microalgae (Delaporte *et al.*, 2005; Parrish, 2009). Several studies have stressed the central roles of such PUFA in the bivalves’ growth (Langdon and Waldock, 1981; Knauer and Southgate, 1997) and gametogenesis (Parrish, 2009). Both species showed similar spatiotemporal variations in the accumulation of ARA and DHA in gills. In contrast, EPA was present at higher proportions in DG and presented almost constant levels in gills with differences of 0.2 to 0.8%, whereas EPA showed important differences in the DG between February and August. This suggests a strong regulation of EPA, which agrees with observations of a moderate incorporation of this FA in the clam *Ruditapes philippinarum* and the oyster *Crassostrea gigas*, even when it was present in high amounts in the food sources (Caers *et al.*, 1998; Delaporte *et al.*, 2005). Another example of regulation from reserve lipids from the DG to gill membrane lipid could be observed for 18:4n-3, which was found at <1% in gill membrane lipids and in proportions higher than 5% in reserve lipids of DG. Not only the spatial and seasonal abundance differences of DHA, EPA and ARA in gills compared to DG but also for 18:1n-9, 18:4n-3 and 18:3n-3 highlight the influence of diet shifts in the physiological regulation of individuals. However, beyond trophic shifts, the FA composition of gills appeared to respond also to the physiological needs for both species. The regulations of long chain FA in membrane lipids have been described during bivalve gametogenesis and are directly related to the diet quality in terms of FA (Soudant *et al.*, 1996; Soudant *et al.*, 1996). Both *N. subnodosus* and *S. crassisquama* share the same period of gametogenesis in the lagoon of Ojo de Liebre, starting around August and finishing in October (Arellano-Martinez *et al.*, 2004; Villalejo-Fuerte *et al.*, 2005). The specific incorporations and/or regulations of these long-chain FA during gametogenesis, associated to the food availability, could be a response to their physiological needs during the reproductive period.

FA regulation of gill membranes depends on the physiological requirements of individuals to cope with changes in environmental variables, such as salinity, dissolved oxygen or temperature (Delaunay *et al.*, 1993; Soudant *et al.*, 1998; Kraffe *et al.*, 2004; Parent *et al.*, 2008)**.** Temperature is the abiotic factor that undergoes the largest range of variation (~6°C between January and August, at both stations) in the lagoon compared to oxygen (variation of ~1 mg.L^−1^ between January and August, at both stations; pers. comm., Reserva de la Biosfera El Vizacaino). Several studies described the importance of temporal modifications in the membrane lipid composition of bivalves, linked to temperature changes (Pernet *et al.*, 2007; Munro and Blier, 2015). In February (winter), when temperatures were lowest in the lagoon (18.6°C at station 1 and 19.4°C at station 2), PUFA increased markedly conversely to total SFA and MUFA, principally due to the increase of DHA, and induced an increase in the overall index of unsaturation. This increase in the overall degree of unsaturation in February suggests that similar factors were driving membranes FA composition at both stations, and for both species. Several studies revealed that while lower temperature reduces the fluidity of cell membranes, higher index of unsaturation contributes to maintain membrane performances at lower temperatures (Hazel, 1995; Ballweg and Ernst, 2017; Martin-Creuzburg *et al.*, 2019), and that lipid remodeling was principally induced by an accumulation of DHA during a decrease in temperature (Pernet *et al.*, 2007; Parent *et al.*, 2008). Although the temperature variation in this tropical lagoon (~6°C between winter and summer) is less important than in the studies by Pernet *et al.* (2007) and Parent *et al.* (2008) (~20°C between winter and summer in sub-Artic zone), membrane lipid compositions seemed regulated following the same key processes of thermal acclimation.

Because *cis* double bonds introduce a kink into the acyl chain, unsaturated FAs pack less compactly and thus can balance the increase in membrane lipid order caused by a decrease in temperature reflecting ability of local adaptation to environmental temperatures in *S. crassisquama* and *N. subnodosus*. Nevertheless, modification of overall membrane unsaturation and regulation of DHA may not be the only purpose of membrane FA restructuring during thermal adaptation. The modulation of lipid fluidity cannot explain why *N. subnodosus* and *S. crassisquama* accumulate PUFA rather than MUFA in their membrane lipids at lower temperatures in February since MUFAs are superior to PUFAs with respect to the magnitude (expressed on a per double bond basis) of the changes in fluidity they produce (Williams and Hazel, 1992). As suggested by other studies on ectotherms, other aspects of membrane organization, such as the modification of lipid class and sub-class composition (e.g. PE/PC ratio, plasmalogen proportion), can regulate membrane function more than simple changes in lipid unsaturation and overall fluidity (Lee, 1991; Hazel, 1995; Kraffe *et al.*, 2007) as discussed below.

### Seasonal comparison of particular bio-synthesize FAs

Whereas both species presented similar spatiotemporal regulations of EFA, a higher proportion of DMA, synthesized through the plasmalogen transesterification reaction, was found for *S. crassisquama*, in February at both stations, suggesting an increase in plasmalogens in gill membrane lipids. In *N. subnodosus*, DMA showed much lower differences, with no seasonal difference in DMA proportions found at station 1, and a small increase in DMA in February at station 2. Although the role of plasmalogens is still few known, especially in marine bivalves, they are supposed to have many biological roles (Nagan and Zoeller, 2001). Higher levels of plasmalogens could reveal one example of a physiological advantage to maintain cell integrity in response to different abiotic stresses, such as temperature (Roots, 1968; Kraffe *et al.*, 2007), oxygen (Zoeller *et al.*, 1999; Le Grand *et al.*, 2014) or salinity variations (Chapelle, 1987). Due to the non-lamellar conformation of the membrane lipids, a high plasmalogen content has already been demonstrated to increase the membrane fluidity and could thus be involved in membrane enzyme activity and membrane dynamics (Hazel and Williams, 1990; Glaser and Gross, 1994). Plasmalogens could also be involved in the immune response of hemocytes and to improve the resistance of bivalves to pathogens (Paltauf, 1983; Le Grand *et al.*, 2011) or cancers (Le Grand *et al.*, 2013, 2014) and more generally in resistance to biotic stresses. Their increasing content during colder seasons could therefore provide *S. crassisquama* a better physiological and immune responses compared to *N. subnodosus*. The enrichment in plasmalogens found in February for *S. crassisquama* could also play a key role in the prevention of membrane oxidation and be implicated in aging by delaying cell senescence (Munro and Blier, 2012, 2015). The vinyl-ether bond of plasmalogens is highly susceptible to oxidative cleavage and rapidly reacts and traps singlet oxygen, free-radicals and other ROS, protecting surrounding PUFA from peroxidation (Leray *et al.*, 2002). With these considerations on plasmalogen levels and functions, this suggests that *S. crassisquama* would respond more efficiently to biotic and abiotic stress than *N. subnodosus*.

Another contrasted pattern of regulations of membrane lipids between both species gills was also found for NMI FAs, 22:4n-9*trans* and 20:1n-11. These modifications could contribute to the structural and functional regulations of membranes as NMI FAs were suggested to confer a resistance to tissues during microbial attack (Paradis and Ackman, 1977) and during temperature changes (Pirini *et al.*, 2007) and also provide protection against oxidation (Kraffe *et al.*, 2004; Delaporte *et al.*, 2005; Barnathan, 2009; Le Grand *et al.*, 2013). The 20:1n-11 and 22:4n-9*trans* were associated to NMI FA to confer resistance and similar functions as NMI to tissues (Kraffe *et al.*, 2010; Le Grand *et al.*, 2011). Because NMI, 22:4n-9*trans* and 20:1n-11 FA were absent from the potential food sources such as particulate organic matter and sedimentary organic matter (Mathieu-Resuge *et al.*, 2019a), their presence in the bivalves tissues is most likely explained by *de novo* synthesis. Indeed, bivalves can *de novo* synthesize NMI FA, due to an active and specific FA elongation and desaturation systems in mollusks (Zhukova, 1991; Kraffe *et al.*, 2004), with MUFA 18:1n-9 and 16:1n-7, precursors for the i and j series of NMI FA (Zhukova, 1986, 1991). In August, the amount of 18:1n-9 in gill membrane lipid and GD reserve lipids from *N. subnodosus* increased significantly at station 2, but levels of both 20:2i and 22:2i decreased, highlighting an important regulation or dysregulation between precursors and NMI contents. In contrast, in *S. crassisquama*, NMI FAs were more abundant in August than in February at both sites. For this species, NMI FA increase in August was principally due to higher proportions of 20:2i and 22:2i and was correlated to higher proportions of n-9 MUFA (18:1n-9 and 20:1n-9) at the same period in gill membrane and DG reserve lipids. As for plasmalogens (DMA), these differences of regulations of NMI FA between species suggest different strategies or capacities to cope with environmental conditions. The same conclusion can be made for 20:1n-11 and 22:4n-9trans that were associated to NMI FA to confer similar functions as NMI to tissues (Kraffe *et al.*, 2010; Le Grand *et al.*, 2011). Indeed, although 20:1n-11 markedly increased in individuals of *S. crassisquama* in August as for NMI, this was much less pronounced for *N. subnodosus*, and 22:4n-9trans was at lower proportions.

In conclusion, the present study showed that the FA composition of bivalve gills cell membranes is affected by both dietary inputs and the physiological requirements under a variable environment, showing specific responses between the two studied species. These results highlight the importance of FA transfers regulation between reserve tissues (DG reserve lipids) and gill cells membrane lipids. Although regulation capacity of both species was evidenced, contrasted trophic environments were reflected in the composition of cell membranes, which tend to moderate the paradigm that only reserve lipids reflect trophic inputs. Even when both species displayed comparable responses to spatiotemporal variability, they showed some specificities in terms of regulations, especially regarding plasmalogens and plasmalogens-associated FA. Seasonal differences of regulation of particular FA and plasmalogens between species may indicate different fitness between these two species in the lagoon of Ojo de Liebre and can be a relevant factor of a healthy population in an ecosystem. Further studies on the regulation capacities in the lipid composition of cell membranes are needed to better understand and predict their importance in the ecological success of different species in a changing environment. Nevertheless, the first results of this study let us think that the organization of cell membranes can be an important factor of fitness differences that lead to the declining stocks of *N. subnodosus*, concomitant to the proliferation of *S. crassisquama* in the lagoon. In a context of global changes and modifications of food resources, this study supports the importance of combining the understanding of trophic factors with physiological approaches, which could be described as ‘trophic ecophysiology’.

## Funding

This work was supported by the Evaluation orientation de la Coopération Scientifique–Asociación Nacional de Universidades e Instituciones de Educación Superior (ECOS-ANUIES) program (PROPHYMUS project 262983); the ‘Laboratoire d’Excellence’ LabexMER (grant number ANR-10-LABX-19); Conacyt-Foins (grant 296397); co-funded by a grant from the French government under the program ‘Investissements d’Avenir’, and two ‘Mission Longue Durée’ received by Edouard Kraffe in CIBNOR from the Institut de Recherche pour le Développement (IRD). The French Research Ministry and Region Bretagne provided Mathieu-Resuge PhD fellowship.
